# Smartphone addiction habit is positively associated with coronary artery disease and its severity in Chinese adults: a case-control study

**DOI:** 10.3389/fcvm.2024.1374797

**Published:** 2024-08-26

**Authors:** Jun Zhu, Sibo Wang, Yujie Wu, Lingfeng Gu, Yao Ma, Yaxin Wang, Liansheng Wang

**Affiliations:** ^1^ Department of Cardiology, Geriatric Hospital of Nanjing Medical University (Jiangsu Province Geriatric Hospital), Nanjing, China; ^2^ Department of Cardiology, The First Affiliated Hospital of Nanjing Medical University, Nanjing, China

**Keywords:** smartphone addiction, coronary artery disease, hypertension, type 2 diabetes mellitus, sleep quality

## Abstract

**Background:**

Coronary artery disease (CAD) has a high incidence and poor prognosis worldwide. It has been confirmed that smartphone addiction (SA) habit can increase the incidence of hypertension and obesity in adolescents. However, the association of SA with CAD and its severity in Chinese adults remains largely unknown.

**Methods:**

A total of 700 Chinese adults (aged 18–70 years) including 350 CAD patients and 350 control subjects were enrolled. The Smartphone Addiction Scale Short Version (SAS-SV) was used to measure SA habit, and the Pittsburgh sleep quality index (PSQI) was used to assess sleep quality. Multiple logistic regression was employed to analyze the relationship between SA habit and CAD.

**Results:**

After adjusting for age, smoking, hypertension, type 2 diabetes mellitus, and other risk factors, there was a significant association between SA habit and CAD in adults (*p* < 0.001). Subgroup analysis showed that there were statistical differences in the correlation between SA habit and CAD in the hypertension, ≤55 years age old, and female subgroups. Moreover, we performed a subgroup analysis based on the number of coronary artery lesions. The result showed that the rate of SA habit in the three-vessel disease group was the highest (*p* < 0.001). We applied Gensini score to evaluate the severity of coronary artery lesions (median Gensini score, 34) and divided all CAD patients into high Gensini score group (>34) and low Gensini score group (≤34), respectively. Compared with low Gensini score group, patients in high Gensini score group were more likely to have SA habit (*p* = 0.049).

**Conclusions:**

There is a positive association of SA habit with CAD and its severity in Chinese adults.

## Graphical Abstract

SA habit was positively associated with CAD and its severity in Chinese adults.

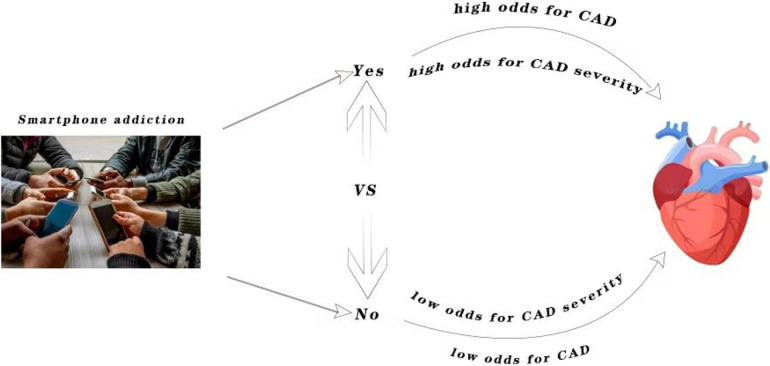

## 1 Introduction

The prevalence of coronary artery disease (CAD) in China is on the rise, causing great threat to society and families (1). The risk factors of CAD include hypertension, type 2 diabetes (T2DM), hyperlipidemia, smoking, obesity and other risk factors. Although hypertension, T2DM and other risk factors have been managed, the current situation of CAD prevention and control is still not optimistic. Therefore, in addition to these traditional cardiovascular risk factors, other risk factors in CAD are worth valuing (2). Discovering new potential risk factors for CAD can benefit the management of CAD and improve the prognosis of CAD patients.

Due to the powerful and attractive advantages of smartphones, many people overuse smartphones, leading to similar addiction symptoms. The definition of smartphone addiction (SA) is psychological dependence on smartphones, referring to long-term dependence on playing with them (3). Just like the essence of the Internet addiction, SA habit is more widespread and hidden, not only among teenagers, but also among adults. Researchers have suggested that excessive use of smartphones may be associated with increased body mass index (BMI), poorer sleep quality and higher hypertension prevalence (4). In addition, patients with SA habit, especially those who prefer to use smartphones before bedtime, often experience changes in sleep habits, are more prone to staying up late, and have various poor sleep quality problems such as short sleep maintenance time, which may be detrimental to physical health (5, 6).

In a quantitative study on SA habit with sleep quality and academic performance in 323 students conducted by Rathakrishnan B et al., it was confirmed that the higher the SA level of college students, the lower their academic performance appeared to be (5). It was also demonstrated that students with poor sleep quality might exhibit lower academic performance. Going to bed late can reduce the short sleep time and lead to poorer sleep quality. Short sleep time or poor sleep quality often lead to insulin resistance and metabolic syndrome by affecting glucose metabolism and blood pressure, thereby increasing the risk of cardiovascular disease (6). Antza C et al. observed the correlation between sleep quality and T2DM, confirming that sleep quality is related to T2DM and obesity (7). Consistent with the above research results, the other two research results also showed that short sleep time would increase the incidence of obesity and metabolic syndrome (8, 9). Ogilvie RP et al. pointed out that short sleep duration and other aspects of sleep deprivation were associated with obesity in both cross-sectional and longitudinal studies. There is a potential causal relationship between sleep deprivation and weight gain, which may be related to the impact of sleep on dietary intake or physical activity (8). By contrast, improving sleep quality contributes to weight control (10). Patients with short sleep time and self-reported sleep problems or sleep disorders have a higher risk of developing hypertension (11). Late sleep duration and high sleep variability are mostly associated with adverse health outcomes in adults (12). In addition, the circadian rhythm disorder caused by late sleep will increase the risk of hypertension, T2DM and obesity. Among people who sleep at night, the incidence rate of T2DM increased 2.5 times, and the incidence rate of hypertension increased 1.3 times (13–15). The decreased secretion of melatonin, changes in sleep structure, or increased sympathetic nervous system activity are considered pathophysiological reasons for the relationship between late sleep and hypertension (13). Late night sleepers are more likely to experience health problems, such as unhealthy eating and sedentary behavior (16, 17). The association between short sleep time and obesity, hypertension, T2DM, and cardiovascular disease may be mediated by changes in dietary intake (16). Patients with SA habit, especially those who prefer to use smartphones before bedtime, are more likely to be exposed to nighttime light, which is associated with cardiovascular disease risk factors including BMI, systolic blood pressure, and low density lipoprotein (LDL) (18). A cohort study confirmed that artificial light while sleeping at night was significantly associated with the risk of increased weight and obesity (19). The mechanisms of light exposure affecting sleep have been confirmed by previous research. Firstly, exposure to light will change the circadian rhythm of sleep (20–22). Secondly, light can directly affect sleep and wakefulness (23). Finally, nighttime lighting can delay some activities that were originally scheduled during the day to nighttime (24). For patients with SA habit who prefer to use their phones before bedtime, excessive exposure to artificial lighting may lead to poorer sleep quality, which increases the risk of developing CAD (25, 26).

In summary, although SA habit may have an impact on CAD related risks such as hypertension, obesity, poor sleep quality, and decreased exercise, there have been no reports on the association between SA habit and CAD patients so far. Therefore, it is necessary to explore the role of SA habit in CAD. This study aims to explore the relationship between SA habit and CAD and its severity in Chinese adults using a case-control study.

## 2 Materials and methods

### 2.1 Study population

**Figure 1:**
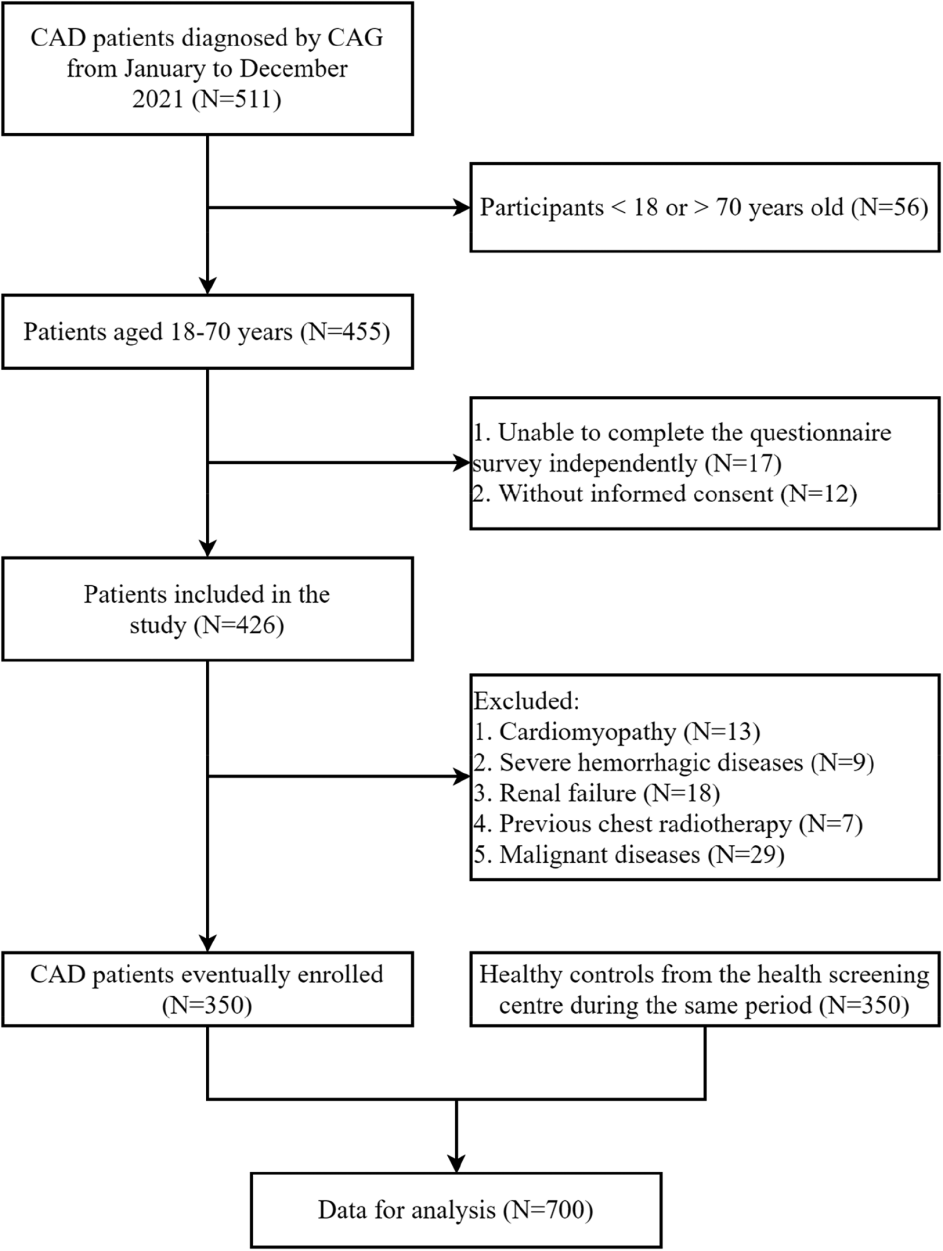
Flow chart of the study population selection. CAD, coronary artery disease; CAG, coronary angiography.

We used statistical methods to calculate the required sample size for this study, and ultimately established that the study included 350 CAD patients and 350 control subjects (aged 18–70 years old) (Figure 1). All selected subjects were unrelated Han Chinese living in or near Jiangsu Province, China. The First Affiliated Hospital of Nanjing Medical University continuously recruited CAD patients. CAD was defined as quantitative coronary angiography using Judkins technology. CAD was defined as angiographic evidence of at least one major coronary artery, including left anterior descending artery, left circumflex artery or right coronary artery, with organic stenosis of more than 50%. Two independent cardiologists who did not know the patient's condition participated in the study and evaluated the angiography. Severity of coronary stenosis was quantified by Gensini score algorithms. The severity of coronary stenosis score was multiplied by the location score of the coronary tree for every lesion, and the sum of all lesions resulted in the Gensini score. It evaluated locations of plaques and numbers of stenotic lesions to estimate the severity of coronary stenosis. The control subjects included 350 patients selected from the same hospital of the health examination center during the same period.

### 2.2 Inclusion and exclusion criteria

Inclusion criteria: (1). CAD group: patients with CAD confirmed by coronary angiography, aged 18–70 years, regardless of sex; (2). Control group: considering that it was immoral to perform coronary angiography to exclude the presence of asymptomatic CAD, the following criteria of 350 control subjects (from the same hospital of the health examination center) were adopted: no history of angina pectoris and no symptoms or signs of other atherosclerotic vascular diseases. Exclusion criteria: Patients who had a history of major concomitant diseases, including cardiomyopathy, hemorrhagic diseases, renal failure, previous chest radiotherapy and malignant diseases, and who were unable to complete the questionnaire independently. The study population consisted of 350 CAD patients and 350 control subjects (Figure 1). With approval from the ethics committee of the First Affiliated Hospital of Nanjing Medical University, written informed consent for the research was obtained from each participant.

### 2.3 Questionnaire

We collected data on age, sex, BMI, hypertension, T2DM, total cholesterol (TC) level, LDL level, triglyceride (TG) level, smoking, alcohol consumption, exercise, arrhythmia, smartphone use, sleep quality, and CAD among mobile phone users. Blood pressure, TC, LDL, TG, and fasting blood glucose were measured during hospitalization. Hypertension is defined as three measurements of blood pressure ≥140/90 mmHg on different days or having received antihypertensive treatment (27). If the fasting blood glucose is ≥7.0 mmol/L, it is defined as T2DM, or if the 2-h blood glucose in the oral glucose tolerance test is ≥11.1 mmol/L, or if the random blood glucose is ≥11.1 mmol/L in addiction to typical T2DM symptoms (if there is no typical symptom of T2DM, it needs to be rechecked and confirmed another day) or hypoglycemic treatment is implemented (28). The Smartphone Addiction Scale (SAS) is a contemporary scale used to assess smartphone usage. The simplified version of the Smartphone Addiction Scale Short Version (SAS-SV) is one of the most widely used tools to measure the degree of SA (29). SAS-SV is a validated scale that includes items rated on 10 dimensions (1 “strongly disagree” to 6 “strongly agree”). The total score ranges from 10 to 60, with the highest score representing the “SAS-SV” of the past year. The items evaluated by the 10 dimensional scale specifically include missing the planned work due to the use of smartphones (item 1); Due to the use of smartphones, it is difficult to concentrate on work (item 2); When using a smartphone, I feel pain in my wrist or back neck (item 3); I can't stand it without a smartphone (item 4); When I don't bring a smartphone, I feel impatient and irritable (item 5); Even if I do not use a smartphone, I will still think of it (item 6); I will never give up using a smartphone, even if my daily life is greatly affected (item 7); I often check my smartphone to avoid missing conversations with others on “WeChat, QQ, or Weibo” (item 8); The time spent using a smartphone is longer than I expected (item 9); People around me tell me that I use smartphones too much (item 10). The content validity, concurrent validity, and internal consistency of the original SAS-SV were all brought into investigation. Kwon stated that the SA thresholds for male and female participants were ≥31 and ≥33, respectively (30). We have revised item 8 of the original SAS-SV to “Frequently check my smartphone to avoid missing conversations with others on Twitter or Facebook”, and replaced “Twitter or Facebook” with “WeChat, QQ, or Weibo” to better align with our local learning audience considering that “WeChat, QQ, and Weibo” are widely used messaging and leisure applications in China. The Chinese version of SAS-SV has consistency internally, ensuring the feasibility of the scale (31).

The Pittsburgh sleep quality index (PSQI) including 19-item self-reported questionnaire was used to evaluate subjective sleep quality over the past month (32). These 19 items were divided into seven components: sleep quality, sleep latency, sleep duration, sleep efficiency, sleep disorders, sleep medication use, and daytime dysfunction. The total score of these seven parts is the PSQI total score. The score range is from 0 to 21. The higher the score, the worse is the sleep quality last month. A PSQI total score of >7 is defined as' poor sleep quality ‘, which has been confirmed in the Chinese population (33). Both SAS-SV and PSQI scales were surveyed and collected with the guidance and assistance of clinical psychologists at the First Affiliated Hospital of Nanjing Medical University (Jiangsu Provincial People's Hospital).

The Gensini score reflected the severity of CAD and was calculated based on the location and severity of coronary artery stenosis. The Gensini score was used to evaluate the severity of CAD. The Gensini score: (1) Coronary stenosis score: ≤25% is 1, 26%–50% is 2, 51%–75% is 4, 76%–90% is 8, 91%–99% is 16, and 100% is 32; (2) Vascular coefficient of lesion location: left main artery × 5. Left anterior descending branch proximal section × 2.5, middle section × 1.5, far section × 1. First diagonal support (D1) × 1. Second diagonal support (D2) × 0.5; Left circumflex proximal segment × 2.5, medium and far sections × 1. Blunt edge branch × 1. Rear support × 0.5; The proximal, middle, and distal segments of the right coronary artery, as well as the posterior descending and right ventricular branches × 1; (3) Integral of the degree of stenosis in various coronary lumens × the sum of the vascular coefficients where the lesion is located. The median Gensini score was 34, and the high group was defined as Gensini score >34, with 164 CAD patients; In the low group, there were 186 CAD patients with a Gensini score of ≤34.

### 2.4 Statistical analysis

The normality of numerical variables was analyzed using Kolmogorov-Smirnov test. Continuous variables including age, BMI, TC, TG, LDL were all normally distributed based on Kolmogorov-Smirnov test and presented as mean values ± standard deviation, whereas categorical data were expressed as number (percentage). The differences in variables between the two groups were compared using Student's *t*-test or chi-square test as appropriate. Variables with significant distribution difference at baseline or closely related to CAD entered in the multivariable logistic regression analysis. Multivariable logistic regression analysis was used to test the relationship between SA and CAD distribution and CAD severity. If *p* value < 0.05, the difference was considered statistically significant. SPSS version 19.0 (Chicago, Illinois, USA) was used for all data analysis.

## 3 Results

### 3.1 Baseline information

The baseline characteristics of the study participants are shown in Table 1. Of the 700 patients, 460 (66%) were male and 240 (34%) were female. The proportions of SA and non SA in the total population were 23.6% and 76.4%, respectively. Compared with non-CAD patients, CAD patients were more likely to be male, to have a history of smoking, alcohol consumption, hypertension, T2DM, increased levels (TC, LDL, TG), increased arrhythmia, decreased exercise, poor sleep quality, and SA habit (all *p* < 0.05). However, there was no statistical difference of BMI (25.10 ± 3.06 vs. 24.77 ± 3.90) between CAD and non CAD groups.

**Table 1:** Baseline characteristics of the study patients.

Characteristics	CAD group (*n* = 350)	Non CAD group (*n* = 350)	*p*
Age (years)	62.06 ± 7.78	58.63 ± 9.57	<0.001
Sex (male), *n* (%)	273 (78.0%)	187 (53.4%)	<0.001
BMI (kg/m^2^)	25.10 ± 3.06	24.77 ± 3.90	0.194
Hypertension, *n* (%)	203 (58%)	154 (44%)	<0.001
T2DM, *n* (%)	71 (20.3%)	35 (10%)	<0.001
Smoking, *n* (%)	123 (35.1%)	88 (25.1%)	0.004
Drinking, *n* (%)	98 (28%)	70 (20%)	0.012
TC (mmol/L)	4.36 ± 1.05	3.98 ± 0.89	<0.001
TG (mmol/L)	1.62 ± 0.86	1.48 ± 0.75	0.020
LDL (mmol/L)	2.88 ± 0.82	2.52 ± 0.71	<0.001
Exercise, *n* (%)	105 (30%)	140 (40%)	0.005
Cardiac arrhythmia, *n* (%)	81 (23.1%)	46 (13.1%)	0.001
Poor sleep quality, *n* (%)	85 (24%)	52 (14.9%)	0.002
Smartphone addiction, *n* (%)	102 (29.1%)	63 (18%)	0.001

CAD, coronary artery disease; BMI, body mass index; T2DM, type 2 diabetes; LDL, low density lipoprotein; TC, total cholesterol; TG, triglyceride.

Age, BMI, TC, TG, LDL (expressed as mean ± SD) were normally distributed and compared by student's *t*-test. Other data was evaluated by Chi-square test. Sleep quality was evaluated using the Pittsburgh sleep quality index (PSQI), and a score greater than 7 was considered poor sleep quality.

### 3.2 Multivariable analysis

Variables such as age, sex, LDL, hypertension, T2DM, exercise, sleep quality, and SA habit were included in the multivariate analysis through a logistic regression model (Table 2). The results showed that SA habit (adjusted OR = 2.17, 95% CI = 1.45–3.26, *p* < 0.001) was significantly associated with CAD in adults.

**Table 2:** Factors affecting CAD by multiple logistic regression analysis.

Variable	OR	95% CI	*p*
Age	1.05	1.03–1.07	<0.001
Sex (male)	2.77	1.93–3.98	<0.001
Hypertension	1.65	1.17–2.32	0.004
T2DM	1.86	1.14–3.03	0.013
Cardiac arrhythmia	2.42	1.52–3.85	<0.001
TC	1.56	1.30–1.87	<0.001
Exercise	0.50	0.34–0.72	<0.001
Poor sleep quality	1.85	1.22–2.80	0.004
SA	2.17	1.45–3.26	<0.001

T2DM, type 2 diabetes; SA, smartphone addiction; CAD, coronary artery disease; LDL, low density lipoprotein.

### 3.3 Subgroup analysis of SA related to CAD patients

**Figure 2:**
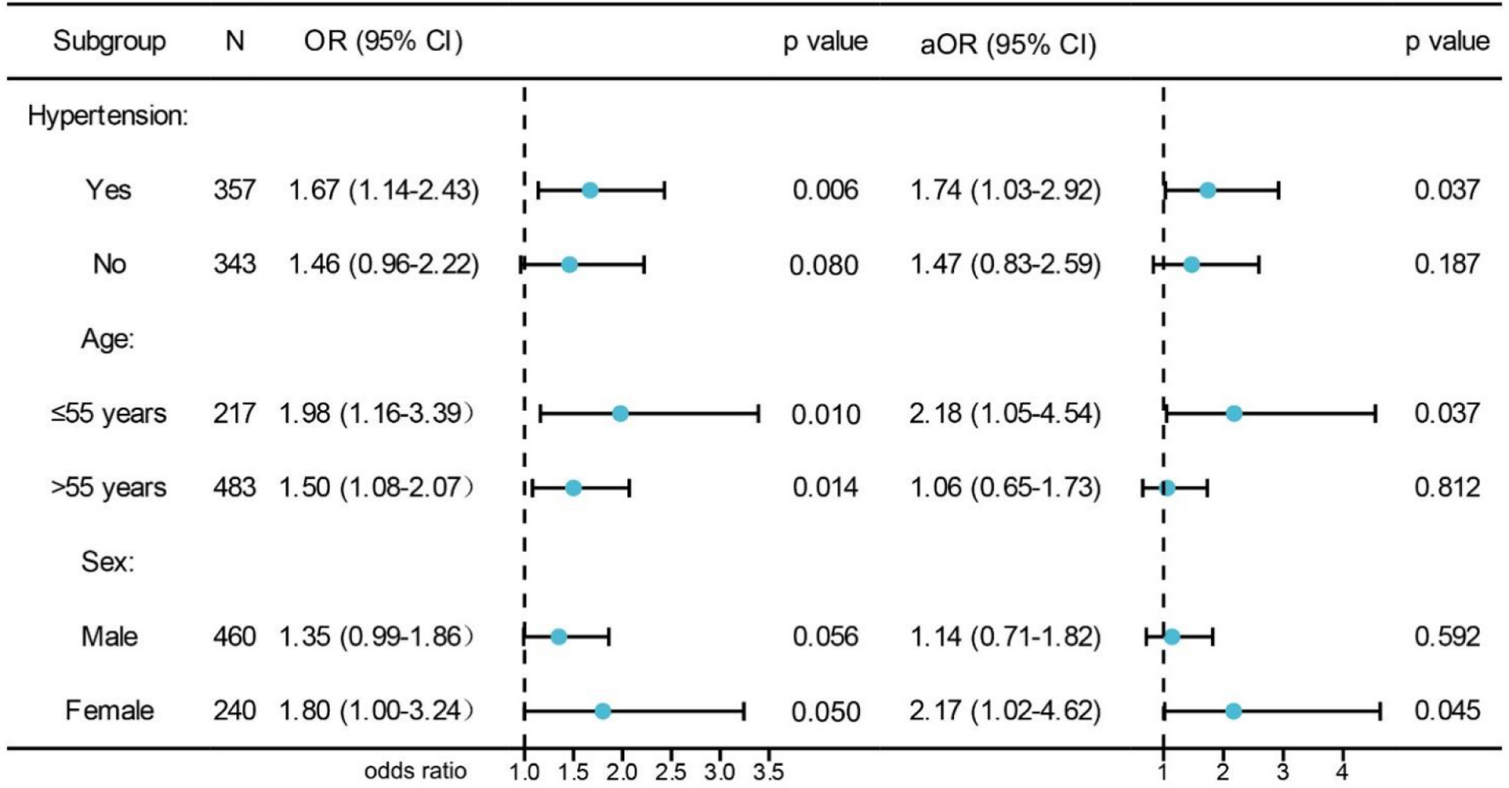
Subgroup analysis of the association between SA and CAD. aOR indicates adjusted OR calculated using multiple logistic regression.

We conducted subgroup analysis stratified by hypertension (yes or no), age (>55 or ≤55 years), and sex (male or female) to further clarify the role of SA in CAD. The results showed that statistical differences were observed between SA and CAD in the hypertension subgroup (adjusted OR = 1.74, 95% CI = 1.03–2.92, *p* = 0.037), age ≤55 years (adjusted OR = 2.18, 95% CI = 1.05–4.54, *p* = 0.037), and female subgroups (adjusted OR = 2.17, 95% CI = 1.02–4.62, *p* = 0.045), while no statistical differences were observed in the non-hypertension subgroup (adjusted OR = 1.47, 95% CI = 0.83–2.59, *p* = 0.187), age >55 years old (adjusted OR = 1.06, 95% CI = 0.65–1.73, *p* = 0.812) and male subgroups (adjusted OR = 1.14, 95% CI = 0.71–1.82, *p* = 0.592) (Figure 2). We divided all enrolled individuals into SA and non SA groups based on the presence of SA and conducted a comparative analysis of baseline data on BMI, smoking, exercise, and sleep quality for all patients in the SA and non SA groups. The results showed that there were statistically significant differences in smoking (*p* = 0.017), decreased exercise (*p* = 0.002), TC (*p* < 0.001), LDL (*p* = 0.001), poor sleep quality (*p* = 0.017) between these two groups (Table 3). However, there were not significant differences in increased BMI (*p* = 0.812) and TG level (0.842). Patients with SA were more likely to experience reduced exercise, smoking, and poor sleep quality. At the same time, we conducted a subgroup analysis based on the number of coronary artery lesion branches. The results showed that there were statistical differences in the distribution of SA habit among CAD populations with different degrees, and SA habit was more pronounced in the triple vessel lesion group (*p* < 0.001). Therefore, SA was related to the severity of coronary artery lesions. We further evaluated the role of SA in the severity of CAD using the Gensini score (the median Gensini score was 34). Then, based on the median Gensini score, we divided CAD patients into high Gensini score group (>34) and low Gensini score group (≤34). In comparison with the low Gensini score group, patients in the high Gensini score group, were more likely to be older, have smoking history, drinking history, hypertension, T2DM, increased levels (TC, LDL, TG) and SA habit (*p* < 0.05) (Table 4). Variables such as age, drinking history, hypertension, T2DM, TC, poor sleep quality, exercise and SA habit were entered into multivariate analysis through a logistic regression model (Table 5). The result showed that SA habit (adjusted OR = 1.66, 95% CI = 1.01–2.76, *p* = 0.049) was significantly associated with high Gensini score group patients (Graphical Abstract).

**Table 3:** Comparison of data between SA habit group and non SA habit group.

Characteristics	SA subgroup	Non-SA subgroup	*p*
All patients (*n* = 700)	165	535	
BMI (kg/m^2^)	24.9 ± 3.00	24.9 ± 3.65	0.812
TC (mmol/L)	4.43 ± 1.18	4.09 ± 0.91	<0.001
TG (mmol/L)	1.56 ± 0.93	1.55 ± 0.77	0.842
LDL (mmol/L)	2.89 ± 0.89	2.65 ± 0.75	0.001
Smoking, *n* (%)	62 (37.6%)	149 (27.9%)	0.017
Exercise, *n* (%)	41 (24.8%)	204 (38.1%)	0.002
Poor sleep quality, *n* (%)	43 (26.1%)	94 (17.6%)	0.017
CAD subgroup (*n* = 350)	102	248	<0.001
Single vessel lesion group	32 (31.3%)	119 (48%)	
Double vessels lesion group	39 (38.3%)	86 (34.7%)	
Triple vessels lesion group	31 (30.4%)	43 (17.3%)	

SA, smartphone addiction; CAD, coronary artery disease; BMI, body mass index; TC, total cholesterol; TG, triglyceride; LDL, low density lipoprotein.

Continuous variables, including BMI, TC, TG, LDL (expressed as mean ± SD), were all normally distributed and compared using Student's-test. Categorical data evaluated by Chi-square test. Sleep quality was evaluated using the Pittsburgh sleep quality index (PSQI), and a score greater than 7 was considered poor sleep quality.

**Table 4:** Correlation between SA habit and severity of CAD using Gensini score.

Characteristics	High Gensini score group 164	Low Gensini score group 186	*p*
Age (years)	63.77 ± 7.59	60.54 ± 7.64	<0.001
Sex (male), *n* (%)	134 (81.7%)	139 (74.7%)	0.117
BMI (kg/m^2^)	25.22 ± 3.24	24.99 ± 2.89	0.483
T2DM, *n* (%)	48 (28.7%)	23 (12.4%)	<0.001
Smoking, *n* (%)	78 (47.6%)	45 (27%)	<0.001
Drinking, *n* (%)	59 (36%)	39 (21%)	0.002
TC (mmol/L)	4.64 ± 1.13	4.12 ± 0.90	<0.001
TG (mmol/L)	1.71 ± 0.77	1.55 ± 0.71	0.049
LDL (mmol/L)	3.15 ± 0.78	2.65 ± 0.80	<0.001
Hypertension, *n* (%)	114 (69.5%)	89 (47.9%)	<0.001
Exercise, *n* (%)	47 (28.7%)	58 (31.2%)	0.608
Poor sleep quality, *n* (%)	42 (25.6%)	43 (23.1%)	0.594
SA, *n* (%)	57 (34.8%)	45 (24.2%)	0.030

CAD, coronary artery disease; BMI, body mass index; T2DM, type 2 diabetes; LDL, low density lipoprotein; TC, total cholesterol; TG, triglyceride; SA, smartphone addiction.

The high Gensini score group had a Gensini score of >34, while the low Gensini score group had a Gensini score of ≤34.

Age, BMI, TC, TG, LDL (expressed as mean ± SD) were normally distributed and compared by Student's-test. Other data was evaluated by Chi-square test. Sleep quality was evaluated using the Pittsburgh sleep quality index (PSQI), and a score greater than 7 was considered poor sleep quality.

**Table 5:** Factors affecting severity of CAD using Gensini score by multiple logistic regression analysis.

Variable	OR	95% CI	*p*
Age	1.06	1.03–1.10	<0.001
Drinking	2.48	1.42–4.33	0.001
Poor sleep quality	1.00	0.59–1.69	0.984
T2DM	2.46	1.37–4.42	0.003
Hypertension	1.84	1.15–2.95	0.012
Exercise	0.83	0.49–1.41	0.491
BMI	1.04	0.96–1.12	0.324
SA	1.66	1.01–2.76	0.049

CAD, coronary artery disease; TC, total cholesterol; SA, smartphone addiction.

## 4 Discussion

The main findings of this study were that risk factors for CAD in Chinese adults include age, male gender, hypertension, T2DM, TC, decreased exercise, poor sleep quality, and SA habit. Further analysis also found that SA habit might be related to smoking history, reduced exercise, poor sleep quality, and hyperlipidemia. Therefore, SA habit might be an independent and important predictor of CAD and its related cardiovascular risk factors.

Zou Y et al. conducted a cross-sectional survey using a random cluster sampling method. A total of 2,639 middle school students (1,218 boys and 1,421 girls) aged 12–15 were included, and a significant correlation was observed between SA habit and adolescent hypertension and obesity (4). There were significant differences in SA habit rates among students of different grades, and the SA rate also showed an upward trend with increased weight, which was crucial for hypertension. We observed that patients with SA habit were more likely to experience decreased exercise and hypertension. However, we did not find that SA habit was associated with increased BMI. Overuse of electronic devices, including smartphones, before bedtime can contribute to exposure to blue rich substances at night, which can lead to delayed circadian rhythms and poor physical condition in the morning. The activity of the autonomic nervous system in the heart can significantly increase the users’ heart rate after prolonged use (34). In addition, a study also observed that excessive use of electronic devices such as smartphones in adolescents might lead to a significant circadian rhythm change (35). The mechanisms involved may be severe inhibition of melatonin and delayed circadian rhythm (36).

Compared to patients who were not addicted to smartphones, patients with SA habit were more likely to skip meals, eat unhealthy foods, gain weight, and suffer from sleep disorders (37). Zou Y et al. also observed a correlation between SA habit and poor sleep quality. We used PSQI to evaluate sleep quality and observed a close relationship between SA habit and poor sleep quality. Poor sleep quality and difficulty falling asleep or maintaining a short sleep duration were considered to be negative consequences of SA habit (38, 39). A study has observed the prevalence of SA habit is 42.4% among nurses (40). In the above study, the SA habit is associated with poorer sleep quality and excessive daytime drowsiness among nurses (40). A study on young people found that 75% of youngsters (aged <30 years old) used smartphones before going to bed, which might increase the likelihood of poor sleep quality (41). Being unable to control their smartphone usage even when in bed and worrying about missing information may be the reasons for leaving their smartphones in bed, as they did not want to miss any notifications (42, 43). Al Battashi N et al. also observed that SA habit was associated with sleep problems in adolescents (44). As for the influence of smart phones on sleep quality, in addition to the delay in sleep, there is another possible reason that blue light from smart phones may have a negative impact on the circadian rhythm, leading to negative sleep consequences, such as sleeping later than expected, thus shortening the overall sleep time (45). In conclusion, SA habit was related to poor sleep quality, which was associated with CAD and its related risks such as hypertension, BMI, LDL. Zhang H et al. analyzed the association of PSQI score with blood pressure and hypertension in rural China (33). They enrolled 27,112 individuals aged 18–79 and divided the PSQI score into four parts: <3, 3 -, 6 -, and ≥9. They used a multivariable logistic regression model with hypertension as the dependent variable to analyze, and also completed a meta-analysis to verify the results of the cross-sectional study. The results showed that poor sleep quality was associated with higher hypertension in the general population, which was visible in both males and females. In addition, the risk of hypertension in patients increased with the increase of PSQI score, and the meta-analysis ultimately confirmed that patients with poor sleep quality had significantly higher overall concomitant OR for hypertension. Nedeltcheva AV et al. further explored the relationship between sleep disorders and CAD, and found a connection between sleep disorders and CAD (46). Laugsand LE et al. also confirmed that sleep disorders such as insomnia were closely related to acute myocardial infarction (AMI) (47). In their study, they observed that compared with those who have never experienced such sleep difficulties, people who struggle to fall asleep almost every night had a multiple adjusted risk ratio of 1.45 for AMI, and those who struggle to maintain sleep almost every night had a multiple adjusted risk ratio of 1.30 for AMI. Patients with poor sleep quality were accompanied by circadian rhythm disorders. On the one hand, cardiovascular diseases are often accompanied with regulation of heart rate, blood pressure, and adrenaline (48, 49). Compared with the morning sleep type, the night sleep type was associated with higher resting heart rate, blood pressure, and adrenaline (15, 50). On the other hand, people who go to bed late at night were more likely to lack physical activity and become obese, which were closely related to cardiovascular disease (17). SA users often have the habit of watching their phones at night and prefer to use them before going to bed, which requires the help of screen lighting. Cross-sectional and comparative studies support the impact of artificial light on sleep. People living in electrified communities often sleep later than those without electricity, and in some cases, their sleep time can also be reduced (20, 51). Light patterns affect sleep quality and physical and mental health (52). Exposure to bright light in the day and evening can severely decrease melatonin and somnolence levels, and delay the circadian rhythm (36). Night light intensity is associated with cardiovascular disease risk factors such as BMI, systolic blood pressure, and LDL (18). From a cohort study, artificial light during sleep at night was significantly associated with the risk of weight gain and obesity, especially among women who sleep with a light or television on in their room (19).

Our research results also found an association between SA habit and hyperlipidemia. The correlation between SA and hyperlipidemia was mainly related to TC and LDL levels rather than TG level. In our study, patients with SA were also found to have poor sleep quality in our study, which has been proven to be closely related to hyperlipidemia in some studies (53, 54). Wang D et al. found that compared with participants with good sleep quality, participants with poor sleep quality had significantly higher risk of hyperlipidemia. Poor sleep quality was therefore related to the incidence rate of hyperlipidemia (53). Lemke MK et al. observed that hypercholesterolemia played an important role in the cardiovascular health of truck drivers, and also collected the demography characteristics and sleep variables of the group (54). The results showed that poor sleep quality was closely related to LDL and TC levels in the drivers. Therefore, they suggested that targeted improvement of drivers' sleep quality could curb the risk of arterial atherosclerosis.

Based on the above research confirming the correlation between SA habit and CAD risk factors such as hypertension, BMI, decreased exercise, poor sleep quality, and hyperlipidemia, we conducted a case-control study to investigate the relationship between SA habit and CAD risk in Chinese adults. Our study showed that SA habit was closely related to CAD and its severity. We further conducted subgroup analysis of CAD based on hypertension, age, and sex, and found that there were distribution differences in the hypertension subgroup, ≤55 years old and female subgroups, while this was not the case in the non-hypertension, >55 years old, and male subgroups. The correlation between SA and hypertension has been confirmed in some studies (4), which involved possible mechanisms such as reduced exercise and poor sleep quality. Our study also confirmed that patients with SA may experience decreased exercise and poor sleep quality. Lo K et al. evaluated the correlation between subjective sleep quality and blood pressure or hypertension (55). They conducted a meta-analysis that included 22 studies, and the results showed a significant correlation between poor sleep quality and the likelihood of developing hypertension. People with poor sleep quality had higher average systolic and diastolic blood pressure than normal people. Among middle-aged women in the climacteric period, poor sleep quality and shorter sleep time were related to higher subclinical carotid atherosclerosis (56). Sleep time disorder was considered a modifiable cardiovascular risk factor (57).

To investigate the correlation between SA habit and the severity of CAD in Chinese adults, we divided CAD patients into single vessel, double vessel, and triple vessel lesions based on the number of lesions. The results showed that there were statistical differences in the distribution of SA habit among CAD populations with different degrees, and SA habit was more pronounced in the three-vessel lesion group. We used the Gensini score to quantify the relationship between the severity of coronary artery stenosis and SA. Our study declared that patients with high Gensini score had a higher prevalence of drinking history, hypertension, T2DM, and SA habit. Our study also observed that SA habit increased the risk of factors such hypertension, T2DM, which were associated with the severity of CAD. Therefore, we speculated that SA habit might ultimately participate in the progression of CAD by affecting the risk of hypertension, T2DM.

This study has several limitations. Firstly, the sample of our study was small and from a single center, thereby reducing the testing effectiveness of statistical model. Larger sample and multicenter studies in the future to further confirm the association between SA habit and CAD are needed. Secondly, we did not collect detailed information on smartphone user usage, which were not conducive to explaining the complex relationship between factors such as SA habit and CAD. Thirdly, there was a possibility of selection bias, since we selected patients without history or symptoms of atherosclerotic vascular diseases from the health examination center as controls. Fourthly, given the case-control study design, we could not draw a causal relationship between SA and CAD. Further prospective studies are warranted.

## 5 Conclusions

There was a positive correlation of SA habit with CAD and its severity in Chinese adults. Our findings suggest the important role of SA in the development of CAD. However, these results need further confirmation from prospective studies.

## Data Availability

The raw data supporting the conclusions of this article will be made available by the authors, without undue reservation.
